# Efficacy and tolerability of brivaracetam monotherapy in childhood and juvenile absence epilepsy: An innovative adaptive trial design

**DOI:** 10.1002/epi4.12628

**Published:** 2022-08-04

**Authors:** Thomas Bast, Anne‐Liv Schulz, Florin Floricel, Diego Morita, Jody M. Cleveland, Jan‐Peer Elshoff

**Affiliations:** ^1^ Epilepsy Center Kork Kehl Germany; ^2^ UCB Pharma Monheim am Rhein Germany; ^3^ UCB Pharma Morrisville North Carolina USA

**Keywords:** antiseizure medication, double‐blind placebo‐controlled, electroencephalogram, randomized withdrawal, seizures

## Abstract

**Objective:**

Despite introduction of several antiseizure medications over the past two decades, treatment options for childhood absence epilepsy (CAE) and juvenile absence epilepsy (JAE) remain limited. We report the innovative adaptive design of an ongoing phase 2/3 trial to evaluate efficacy, safety, and tolerability of brivaracetam (BRV) monotherapy in patients 2–25 years of age with CAE or JAE.

**Methods:**

N01269 (ClinicalTrials.gov: NCT04666610; start: July 2021; expected completion: 2024) is a randomized, dose‐finding and confirmatory, double‐blind, placebo‐controlled, parallel‐group, multicenter trial. The trial consists of a dose‐selection and assessment for futility stage, followed by an optimal‐dose stage after interim analysis. Both stages include an up to 2‐week screening period, a 2‐week placebo‐controlled period, and an 11‐week active treatment period (10 weeks of initial treatment followed by a 24‐hour electroencephalogram [EEG] and an additional week of active treatment for 24‐hour EEG assessment). Patients who are absence seizure‐free will enter an up to 4‐week randomized withdrawal period. Efficacy assessments will be based on 24‐hour EEG and seizure diaries.

**Significance:**

This two‐stage adaptive trial design allows investigation of two potentially efficacious BRV doses, where one dose is dropped in favor of the other dose with a better benefit–risk profile. This allows for a combined phase 2 dose‐finding and phase 3 confirmatory efficacy trial, which reduces the number of patients needed to be recruited and reduces trial duration. A randomized withdrawal period is included to evaluate sustainability of treatment effect over time and to allow for placebo control while minimizing placebo exposure. Use of EEG capture in addition to seizure diaries offers a robust mechanism of detecting seizure activity and measuring treatment effect. Positive efficacy and safety/tolerability data may support the use of BRV as monotherapy for CAE or JAE, providing another treatment option and representing long‐delayed progress in the treatment of absence seizures in these populations.

**Key points**

- Treatment options for childhood absence epilepsy (CAE) and juvenile absence epilepsy (JAE) are limited.
- This trial evaluates efficacy, safety, and tolerability of brivaracetam as monotherapy in patients 2–25 years of age with CAE or JAE.
- The adaptive design allows for a combined phase 2 dose‐selection and assessment for futility stage, followed by a phase 3 optimal‐dose confirmatory stage after interim analysis.
- Electroencephalogram capture in addition to seizure diaries offers a robust mechanism of detecting seizure activity and measuring treatment effect.
- Trial design includes randomized withdrawal to show sustainability of treatment effect over time and allows for placebo control while minimizing placebo exposure.

## 1 INTRODUCTION

Childhood absence epilepsy (CAE) and juvenile absence epilepsy (JAE) are classified by the International League Against Epilepsy (ILAE) as genetic/idiopathic generalized epilepsy types, characterized by frequent (multiple daily) brief absence seizures for CAE and less frequent absence seizures (e.g., once daily) for JAE.^1^ Approximately 80% of patients with JAE also have generalized tonic–clonic seizures, which can further complicate finding effective treatments.^2^

There are few trials of antiseizure medication (ASM) treatment for CAE and JAE. In 1996, Trudeau et al assessed the efficacy and safety of gabapentin monotherapy in two small double‐blind, placebo‐controlled trials, followed by open‐label follow‐up.^3^ In 1999, Frank et al investigated the efficacy of lamotrigine (LTG) monotherapy for newly diagnosed typical absence seizures in children and adolescents using a two‐phase “responder‐enriched” trial design, which included an open‐label dose‐escalation period followed by a double‐blind, placebo‐controlled period to assess drug efficacy.^4^ A decade later, Glauser et al assessed the comparative efficacy and tolerability of ethosuximide (ESM), valproic acid (VPA), and LTG in a double‐blind, randomized, controlled trial.^5^ In 2008 and 2011, a long‐term open‐label trial^6^ and randomized placebo‐controlled trial^7^ assessed efficacy of levetiracetam (LEV) in the treatment of typical absence seizures in patients with CAE and JAE.

ESM, VPA, and LTG are commonly used as treatments for CAE and JAE.^8^, ^9^ However, although ESM and VPA are approved treatments of absence seizures in the USA, European Union, and other parts of the world,^10^, ^11^, ^12^, ^13^, ^14^, ^15^ LTG is only indicated for treatment of absence seizures in the European Union,^16^ but not in the USA.^17^ Further limitations of the few approved treatment options include that ESM is not reliably effective against generalized tonic–clonic seizures, limiting its use for patients with JAE in which this type of seizure is common^18^; VPA has important safety issues such as a potentially negative effect on attention and cognitive development, liver and bone marrow toxicity, and teratogenicity, which raises important concerns for the treatment of young females^5^, ^19^, ^20^, ^21^, ^22^; and LTG is less efficacious than VPA and ESM in treating absence seizures, requires prolonged titration because of the risk of severe cutaneous reactions, has a high potential for relevant drug–drug interactions (including oral hormonal contraceptives), and may exacerbate myoclonic seizures.^5^, ^23^ Despite the introduction of newer ASMs over the past two decades, treatment options for CAE and JAE remain limited as none of the newer ASMs have been approved for the treatment of CAE or JAE.

In CAE, approximately one‐third of patients continue to have seizures despite an initial and second therapy, meeting the ILAE criteria for drug‐resistance.^20^ Patients with JAE are less likely to achieve seizure freedom than patients with CAE.^24^ There is a need for additional treatments for patients with CAE and JAE, particularly for monotherapy which may improve treatment adherence and tolerability compared with combination therapies.^25^

Brivaracetam (BRV) is currently indicated for adjunctive treatment of focal seizures in patients 2 years of age and older in the European Union,^26^ and as monotherapy and adjunctive treatment in patients 1 month of age and older in the USA.^27^ BRV is well tolerated, easy to use with no required titration period, and has the potential to be a valuable monotherapy treatment option for patients with typical absence seizures. BRV has shown dose‐dependent efficacy in preclinical studies using genetic absence epilepsy rats,^28^ and in a proof‐of‐concept trial in patients with photosensitive epilepsy.^29^

This is the first placebo‐controlled trial of an ASM in CAE and JAE in more than a decade and reflects current regulatory positions on trial design.^30^ The clinical appearance of absence seizures is more subtle than other seizure types and some absence seizures may not be recognized.^31^ In absence seizures, the ictal and interictal electroencephalogram (EEG) is very characteristic. Therefore, capture of EEG data is critical for seizure diagnosis and to measure the treatment effect. As absence seizures typically occur at short intervals, the effect of ASMs on typical absence seizures can be detected within a few weeks of treatment.^32^ A reduction in absence seizures with LEV (a close structural analog of BRV that shares the same primary mechanism of action^33^) was shown after 14 days of treatment,^7^ and BRV can be initiated at an effective dose without titration. Therefore, assessment of efficacy at 14 days is reasonable. Placebo‐controlled periods longer than 14 days would not be considered ethically acceptable.

The primary objective of this trial is to investigate the efficacy of BRV monotherapy in patients 2–25 years of age with CAE or JAE. The secondary objective is to evaluate the safety and tolerability of BRV monotherapy in patients 2–25 years of age with CAE or JAE.

## 2 METHODS

### 2.1 Trial design

N01269 (ClinicalTrials.gov: NCT04666610) is a phase 2/3, randomized, dose‐finding and confirmatory, double‐blind, placebo‐controlled, parallel‐group, multicenter trial with a two‐stage adaptive design and randomized withdrawal to evaluate the efficacy, safety, and tolerability of BRV as monotherapy in patients 2–25 years of age with CAE or JAE.

Current trial settings are community clinic/academic hospitals in Australia, Belgium, Georgia, Italy, Poland, Romania, Slovakia, Spain, Ukraine, and the USA. The trial started enrolling patients in July 2021 and is expected to complete in 2024.

**FIGURE 1:**
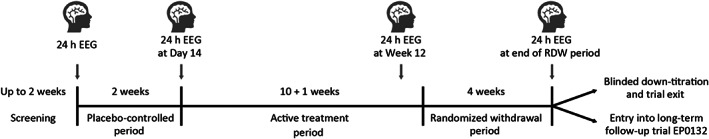
Trial design. EEG, electroencephalogram; h, hour; RDW, randomized withdrawal.

The trial consists of a dose‐selection and assessment for futility stage (Stage 1) followed by an optimal‐dose stage (Stage 2) after interim analysis. The total duration of the trial for a participant is up to 23 weeks. Both stages follow the same overall design (Figure 1): an up to 2‐week screening period, a 2‐week placebo‐controlled (PC) period, and an 11‐week active treatment (AT) period, comprising 10 weeks of initial treatment followed by a 24‐hour EEG and an additional week of active treatment to allow for the assessment of the 24‐hour EEG; patients who are absence seizure‐free on this 24‐hour EEG will enter an up to 4‐week randomized withdrawal (RDW) period. Patients completing the 24‐hour EEG at the end of the RDW period may continue on BRV 100 mg/day in the open‐label, long‐term follow‐up (LTFU) trial (EP0132; ClinicalTrials.gov: NCT05109234) or exit the trial after blinded down‐titration (maximum 2 weeks) and safety follow‐up (2 weeks).

Patients are randomized twice in this trial. Patients are randomized at baseline for treatment during the PC and AT periods, and at entry into the randomized withdrawal period.

In this trial, absence seizures are defined as electrographically recorded symmetric generalized spike waves (2.5‐6 Hz) for 3 seconds or more. Coexisting clinical symptoms are not required for qualifying an EEG event as a seizure.

This trial will enroll patients 2–25 years of age with a diagnosis of CAE or JAE as defined by ILAE criteria (for detailed eligibility criteria, see Supplemental methods in the online Supporting Information). Because response rates to initial and secondary monotherapy have been shown to be similar,^20^ both newly diagnosed (untreated) patients and patients pretreated for absence seizures with a maximum of two historical ASMs (but without ASM treatment for a period of at least five half‐lives of the ASM before randomization into this trial) are eligible for this trial. Eligible patients will be randomized, stratified by syndrome (CAE vs JAE) and by previous treatment for absence seizures (untreated vs treated). Stratification by syndrome will aim to achieve at least 30% of patients within each syndrome. Patient treatment details will be allocated and maintained by an interactive response technology system based on a predetermined production randomization. Unblinding is not allowed except in the event of an emergency (e.g., tonic–clonic seizure during RDW).

#### 2.1.1 Stage 1 (dose‐selection and assessment of futility)

**FIGURE 2:**
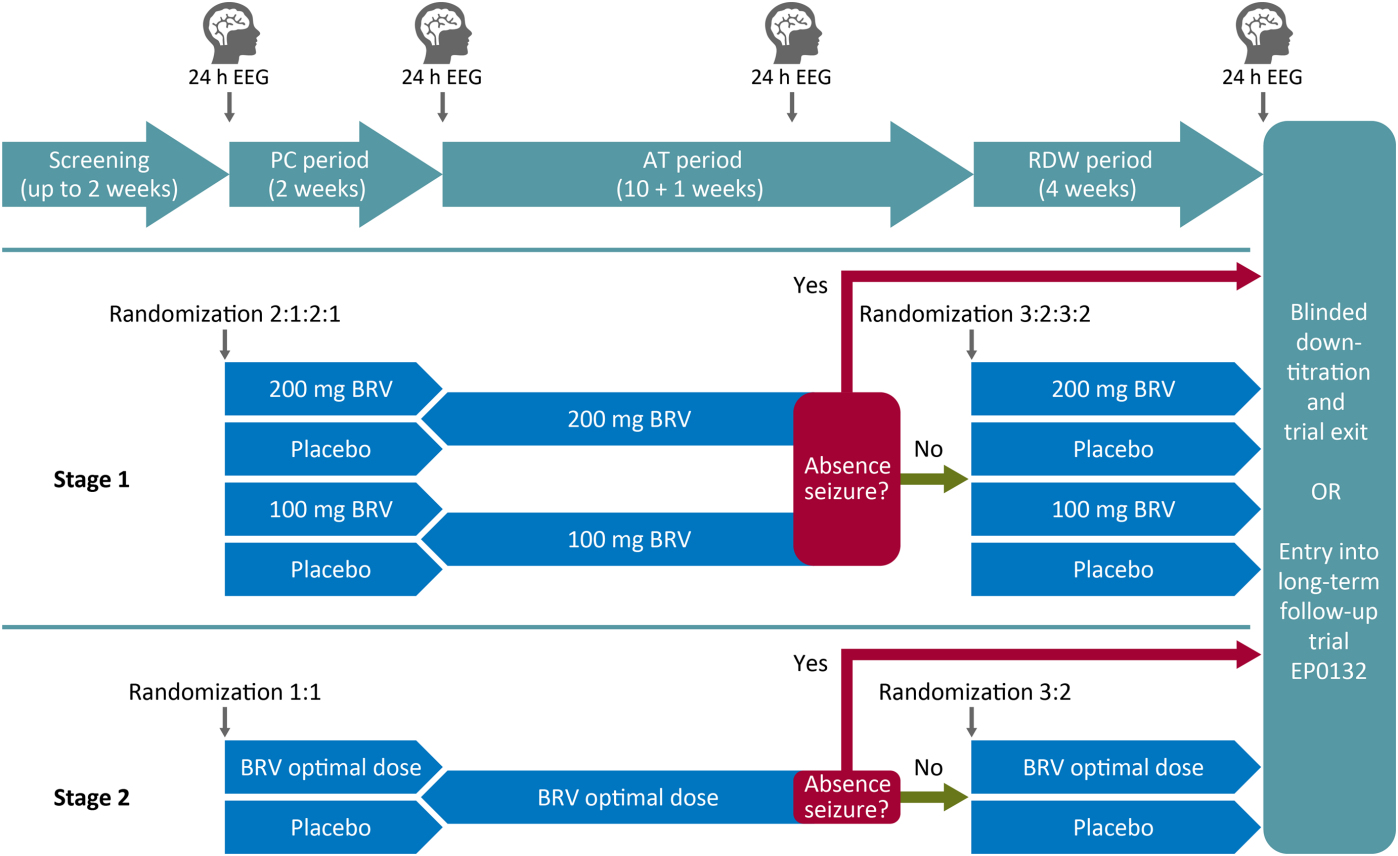
Treatment during Stage 1 and Stage 2. Down‐titration followed by 2 weeks safety follow‐up can occur at any time after first dose of trial drug. 100 mg BRV corresponds to 2 mg/kg/day BRV for patients <50 kg body weight; 200 mg BRV corresponds to 4 mg/kg/day BRV for patients <50 kg body weight. AT, active treatment; BRV, brivaracetam; EEG, electroencephalogram; h, hour; PC, placebo‐controlled; RDW, randomized withdrawal.

Patients will be randomized 2:1:2:1 to receive one of four possible options during the PC and AT periods: (A) BRV 100 mg/day (2 mg/kg/day for patients <50 kg body weight), (B) “placebo to BRV 100 mg/day” (2 mg/kg/day for patients <50 kg body weight), (C) BRV 200 mg/day (4 mg/kg/day for patients <50 kg body weight), or (D) “placebo to BRV 200 mg/day” (4 mg/kg/day for patients <50 kg body weight) (Figure 2). Patients randomized to a placebo group will receive placebo during the PC period and BRV during the AT period. Dose adjustments will not be allowed during the PC or AT periods.

BRV and placebo will be administered as oral solutions taken in equal doses twice daily, 12 hour apart. BRV will be available in three concentrations: 10 mg/ml (equivalent to the commercially available solution), 5 mg/ml, and 2.5 mg/ml solutions. Patients randomized to 200 mg/day (4 mg/kg/day for patients <50 kg body weight) dosing will receive the 10 mg/ml solution, while patients randomized to 100 mg/day (2 mg/kg/day for patients <50 kg body weight) dosing will receive the 5 mg/ml solution. To maintain blinding, the volume of solution to be taken at each dosage will be the same, irrespective of randomized treatment.

When ~25% of patients have completed the trial, blinded safety data will be provided to the Independent Data Monitoring Committee (IDMC; consisting of members with experience and expertise in clinical trials and management of absence seizures in pediatric and young adult patients, as well as an independent statistician) to assess safety. When ~84 patients are evaluable for assessment of efficacy in Stage 1 (assessment for futility and dose‐selection), an interim analysis on Stage 1 efficacy and safety will be performed by an independent statistical team. The results of the interim analysis will be provided to the IDMC who will determine first futility, and then the optimal BRV dose to carry into Stage 2, utilizing results from analyses of Stage 1 efficacy and safety. Patients must have completed the first visit of the AT period to be considered evaluable for the interim efficacy analysis. Enrollment will not stop during the conduct of the interim analysis. The IDMC will continue to monitor safety for the trial duration. Sites and patients will remain blinded to the optimal dose selected for Stage 2.

#### 2.1.2 Stage 2 (optimal dose)

Patients will be randomized 1:1 to receive either a) the optimal BRV dose or b) “placebo to optimal BRV dose” during the PC and AT periods (Figure 2). Patients randomized to “placebo to optimal BRV dose” will receive placebo during the PC period and the optimal BRV dose during the AT period. Dose adjustments will not be allowed during the PC or AT periods.

#### 2.1.3 Randomized withdrawal period

Patients who are absence seizure‐free based on the outcome of the 24‐hour EEG after the Stage 1 or Stage 2 AT period will enter the 4‐week RDW period and will be randomized in a double‐blind fashion in a 3:2 ratio to either continue their BRV dose as received during the AT period or to down‐titrate to placebo (receiving 100 mg/day in Week 1, 50 mg/day in Week 2, and 0 mg in Weeks 3 and 4) (Figure 2). Patients who completed the AT period but show absence seizures on the 24‐hour EEG are ineligible for the RDW period and may instead continue into the LTFU trial on BRV 100 mg/day (2 mg/kg/day for patients <50 kg body weight).

For patients who report absence seizures at any time during the RDW period, a 1‐hour EEG (with hyperventilation) will be obtained. If an absence seizure is observed during this 1‐hour EEG, the patient may leave the trial and enter the open‐label LTFU trial. If no absence seizure is observed during this 1‐hour EEG, the patient will receive a 24‐hour EEG. If an absence seizure is observed during this 24‐hour EEG, the patient may leave the trial and enter the open‐label LTFU trial. If no seizure is detected during this EEG, the patient will continue the RDW period.

Patients with no absence seizure during the RDW period will have a 24‐hour EEG at the end of the RDW period and will be allowed to enter the LTFU trial regardless of the result of this EEG.

#### 2.1.4 Down‐titration and safety follow‐up

**FIGURE 3:**
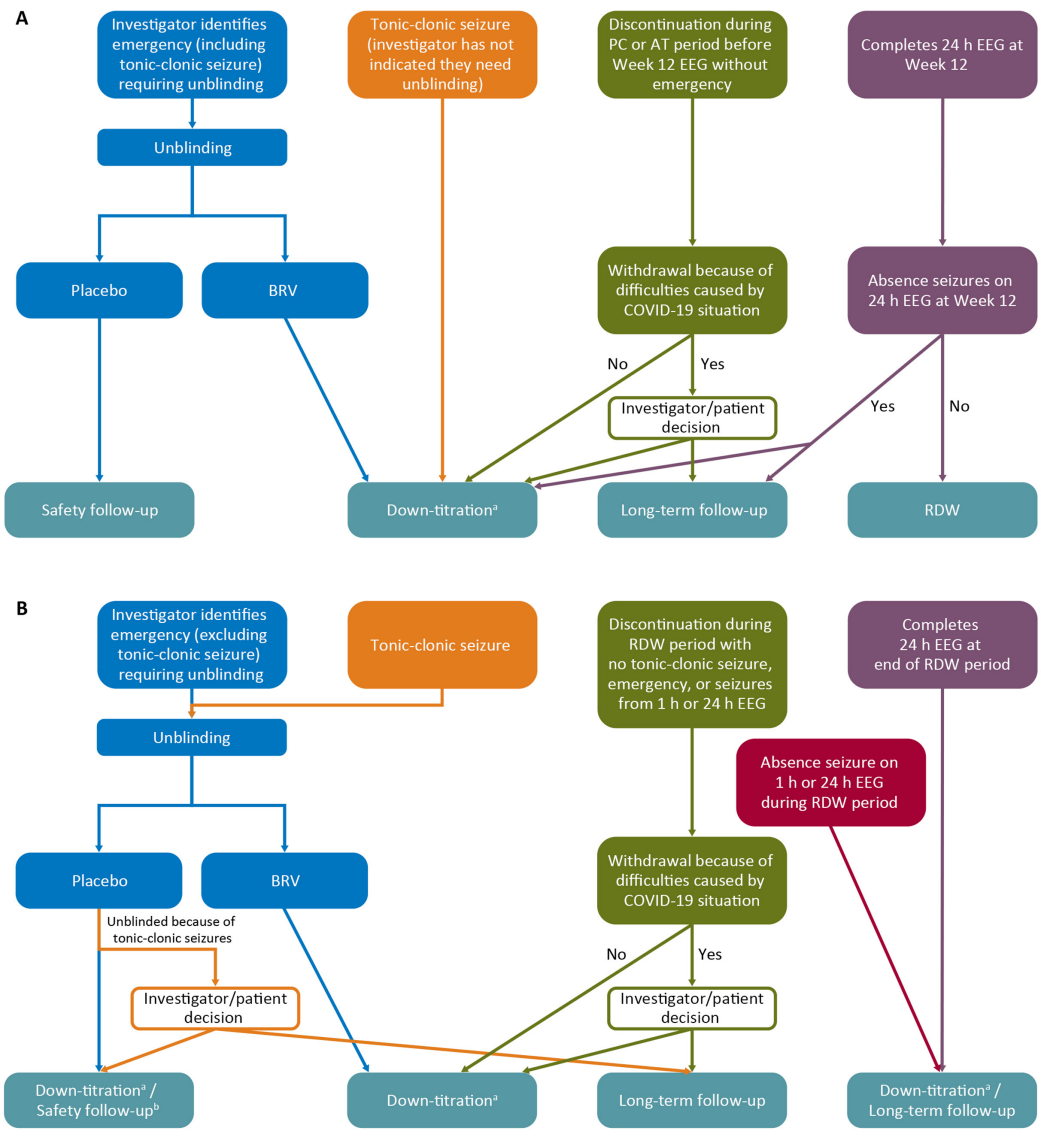
Potential patient scenarios (A) during the placebo‐controlled and active treatment periods or (B) during the randomized withdrawal period. AT, active treatment; BRV, brivaracetam; COVID‐19, coronavirus disease 2019; EEG, electroencephalogram; h, hour; PC, placebo‐controlled; RDW, randomized withdrawal. ^a^A safety follow‐up visit will occur 14 days after the last dose of the down‐titration scheme; ^b^Patient will enter unblinded down‐titration if still on BRV at the time (first 2 weeks of the randomized withdrawal), otherwise the patient will directly enter the safety follow‐up period.

All patients either enter the LTFU trial or are down‐titrated. Different scenarios apply during the PC or AT periods (Figure 3A) and RDW period (Figure 3B).

Patients who experience an emergency requiring unblinding, who experience a tonic–clonic seizure during the PC or AT period (if it is considered an emergency requiring unblinding), or who experience a tonic–clonic seizure during the RDW period (independent of whether it is considered an emergency) will be unblinded. Patients receiving BRV will be withdrawn from the trial after an open‐label down‐titration and safety follow‐up, whereas patients receiving placebo will directly enter the safety follow‐up period. Patients who experience a tonic–clonic seizure during the RDW period will have the option to enter the LTFU trial if unblinding determines they are receiving PBO. Patients who do not meet criteria for unblinded, open‐label down‐titration and do not enter the LTFU trial will follow a blinded down‐titration.

If absence seizures are detected on the 24‐hour EEG at Week 12 during the AT period, or on any EEG during the RDW period, patients may enter the LTFU trial or follow blinded down‐titration.

Some patient scenarios depend on whether patients withdraw because of coronavirus disease 2019 (COVID‐19)‐related difficulties or not. Patients who discontinue during the PC or AT period before the Week 12 EEG without an emergency requiring unblinding or who discontinue during the RDW period with no tonic–clonic seizure, no emergency requiring unblinding, and no absence seizures confirmed by 1‐hour or 24‐hour EEG can enter the LTFU trial or follow blinded down‐titration if they withdraw because of difficulties caused by the COVID‐19 situation. If patients discontinue in these circumstances because of non‐COVID‐19‐related reasons, they will follow a blinded down‐titration and are not allowed to enter the LTFU trial.

During blinded down‐titration, the dose will be BRV 100 mg/day (2 mg/kg/day for patients <50 kg body weight) for Week 1 and BRV 50 mg/day (1 mg/kg/day for patients <50 kg body weight) for Week 2. For unblinded down‐titration, the doses are at the discretion of the investigator. Unblinded down‐titration can last up to 4 weeks, providing additional flexibility. A safety follow‐up visit will occur 14 days after the last dose of the (blinded or unblinded) down‐titration scheme.

Discontinuation of BRV should be considered by the investigator for abnormal liver function, or clinically significant finding (e.g., change from baseline in QT interval), or if it is considered in the best interest of the patient.

### 2.2 Endpoints

Efficacy, safety, and pharmacokinetic (PK) endpoints will be assessed (Table 1). All assessments will be completed at specific trial visits (Table S1). EEG reading will be software‐guided with visual confirmation by the central reader. All adverse events that occur during the trial will be documented. Blood samples for PK assessments will be collected at treatment Weeks 2, 13, and 17.

**TABLE 1:** Trial endpoints

Primary efficacy endpoints Absence seizure freedom within 4 days prior to or during the 24 h EEG at Day 14^a^ Day 14 was chosen based on the efficacy results in an earlier trial of LEV^7^ and to avoid a longer exposure to placebo Primary estimand^b^ used four attributes to define treatment effect: Population: the patient population as defined in the protocol‐specified inclusion/exclusion criteria reflecting the target population for approval Patient‐level outcome: absence seizure freedom within 4 d prior to or during the 24 h EEG at Day 14 Intercurrent event handling: the intercurrent event of concomitant use of any BZD within 4 d prior to or during the 24 h EEG at Day 14 is handled in the definition of the participant level variable implementing a composite strategy in which receiving any concomitant BZD within 4 d prior or during the 24 h EEG at Day 14 is counted as nonresponse (i.e., considered as having absence seizures during the EEG) Population‐level summary measure: conditional odds ratio comparing BRV with placebo
Secondary efficacy endpoints Absence seizure freedom during the RDW period as determined by 24 h EEG at the end of the RDW period Percentage change from baseline to Day 14 in number of absence seizures on 24 h EEG Absence seizure freedom based on diary during the 4 d prior to the visit at Day 14 Absence seizure freedom on 24 h EEG at Week 12 Absence seizure freedom based on diary during the 4 d prior to the visit at Week 12
Other efficacy endpoints Percentage change from baseline to Week 12 in number of absence seizures on 24 h EEG Percentage change from baseline to Week 12 in number of days with absence seizures per 7 d based on diary Change from baseline to Day 14 in percentage time of day with generalized spike waves on 24 h EEG^c^ Change from baseline to Week 12 in percentage time of day with generalized spike waves on 24 h EEG^c^
Exploratory endpoint Time to recurrence of absence seizures based on diary (for patients who were absence seizure‐free in the 4 d prior to Day 14)
Secondary safety and tolerability endpoints TEAEs TEAEs leading to discontinuation of trial treatment Serious TEAEs Trial drug‐related TEAEs
Other safety endpoints Changes from baseline to each applicable visit in safety laboratory tests, vital signs, ECG parameters, body weight, and height ECG findings at each applicable visit Physical and neurological examinations findings at each applicable visit Suicidal risk monitoring for patients ≥6 y of age Patient‐ or parent‐reported outcomes (EpiTrack Junior for patients 6 to <18 y of age at trial entry; PedsQL Generic Core Module for patients ≤17 y) Occurrence of other seizure types, including GTCS based on diary or EEG
Pharmacokinetic Plasma concentrations of BRV

Abbreviations: BRV, brivaracetam; BZD, benzodiazepine; ECG, electrocardiogram; EEG, electroencephalogram; GTCS, generalized tonic–clonic seizure; LEV, levetiracetam; PedsQL, Pediatric Quality of Life; RDW, randomized withdrawal; TEAE, treatment‐emergent adverse event.

^a^ The primary efficacy endpoint incorporates absence seizure occurrences during the 24 h EEG only and BZD usage during the EEG and within 4 d prior to the EEG; absence seizures outside of the 24 h EEG will not contribute to the primary endpoint unless requiring use of BZD; the inclusion of “4 days prior” was incorporated in the primary endpoint to align with the estimand language, which has BZD usage as the intercurrent event for the primary endpoint; to achieve absence seizure freedom, the patient must have no absence seizures in their 24 h EEG and not used BZD within 4 d prior to the EEG.

^b^ For the estimand, the composite strategy has been selected as the primary approach for the intercurrent event handling because the use of concomitant BZD within 5 d of the EEG is assumed to have a significant effect on the EEG efficacy evaluation at Day 14.

^c^ Data will be provided by the EEG vendor as “total duration of generalized spike waves” and “total evaluable hours of EEG while awake and asleep” (in hours, minutes, and seconds); the percentage of generalized spike waves will be calculated as a proportion of the total readable time during the 24 h EEG.

### 2.3 Statistical methods

To achieve a power of 90%, a sample size of 28 patients per arm in each stage would be required. Assuming a 15% rate of nonevaluable patients provides an estimated recruitment sample size of 160 randomized patients (32 patients per arm).

The following analysis sets are planned: All Patients Screened Set (all screened patients with informed consent/assent), Randomized Set (all patients randomized in the PC period), Safety Set (all patients who took at least one dose of trial drug), Full Analysis Set (randomized patients who took at least one dose of trial drug), RDW Analysis Set (all patients randomized in the RDW period who took at least one dose of trial drug during the RDW period), and Pharmacokinetic Per Protocol Set (all patients who took at least one dose of BRV and have at least one valid BRV plasma concentration–time record and dosing information available).

Computations for the non‐PK analyses will be performed using SAS® version 9.3 or later (SAS Institute). All statistical testing will be carried out using a two‐sided 5% significance level unless otherwise indicated. Descriptive statistics will be used to summarize adverse events by treatment group, for plasma concentration data, and for “other” efficacy endpoints.

## 3 DISCUSSION

This trial uses a two‐stage design, which allows investigation of two doses of BRV with the potential to be efficacious for the treatment of absence seizures, where one dose is dropped in favor of the other dose with a better benefit–risk profile. The planned design allows for the combination of a phase 2 dose‐finding and a phase 3 confirmatory efficacy trial, which reduces the overall number of patients needed to be recruited and at the same time reduces the trial duration.

Previous trials of ASMs in children and adolescents with absence seizures did not use a placebo‐controlled and randomized‐withdrawal design, and did not include stratification by previous treatment status and CAE/JAE.^3^, ^4^, ^5^, ^7^ The randomized withdrawal design allows for the benefits of a placebo‐controlled trial (such as comparative design) while minimizing the amount of time patients will need to remain on placebo. The RDW period was included to show sustainability of the effect after 3 months of treatment. This component of the trial design was added after regulatory consultation. The maximum time on placebo treatment will be 14 days in the initial PC period and 14 days in the RDW period. Few patients will experience placebo twice as randomization to BRV or placebo occurs during the PC period (2:1 in Stage 1; 1:1 in Stage 2) and RDW period (3:2). The overall time on placebo is considered acceptable given that patients are often undiagnosed and untreated for weeks or months and a short period without treatment would not affect their long‐term prognosis. Furthermore, in case of absence seizures during the RDW period, patients randomized to placebo can be put back on BRV in the LTFU trial after confirmation of absence seizures.

BRV can be started with a therapeutic dose on Day 1, and a reduction in absence seizures with LEV was shown after 14 days of treatment.^7^ Moreover, patients with CAE or JAE have frequent seizures.^1^ Therefore, an efficacy assessment after 14 days of treatment based on 24‐hour EEG is deemed appropriate to assess the efficacy of BRV in this population. The clinical appearance of absence seizures is more subtle than other seizure types.^31^ The use of EEG data capture in addition to diary recording of absence episodes offers a robust mechanism of detecting seizure activity and measuring treatment effect.

One limitation of the planned trial design is the inclusion of periods where patients with controlled seizures would be subject to a withdrawal of BRV. However, absence seizure recurrences are expected to occur rapidly following withdrawal, with an associated rapid reintroduction of trial drug, reducing the amount of time where untreated absence seizures could occur. Furthermore, absence seizures are generally considered low risk. An additional limitation is that patients with a history of nonfebrile seizures other than absence seizures are excluded from the trial. This may lead to exclusion of patients with JAE who often experience generalized tonic–clonic seizures.^2^ This exclusion criterion was included to not put patients with generalized seizures at unnecessary risk. Moreover, patients experiencing generalized tonic–clonic seizures during the trial will be withdrawn for safety reasons.

BRV is initiated at full dose (100 mg/day in adults and children weighing at least 50 kg) without titration and has been shown to be generally well tolerated in pediatric patients 4–16 years of age with epilepsy and in adult patients with focal seizures.^34^, ^35^, ^36^, ^37^ Safety data generated in trial N01263 (NCT00422422; an open‐label, single‐arm, PK, safety, and efficacy trial of BRV as adjunctive therapy in patients from 1 month to <16 years of age with epilepsy) and its long‐term extension trial, N01266 (NCT01364597; an open‐label, long‐term safety trial of BRV as adjunctive therapy in patients 1 month to <17 years of age) support the tolerability of BRV in patients 4 to ≤25 years of age with CAE or JAE.^36^, ^37^ The inclusion of patients <4 years of age in this trial is in line with the approved age range for BRV in the USA.^27^ In addition, patients 2–4 years of age will not take part in the Stage 1 dose ranging part of the trial.

This trial will provide the first new data of treatment in CAE or JAE in over a decade and will use an innovative trial design including randomized withdrawal to show sustainability of treatment effect over time, and allowing for placebo control while minimizing placebo exposure. Positive efficacy and safety/tolerability data may support the use of BRV monotherapy in the treatment of patients with CAE or JAE, providing another treatment option and representing long‐delayed progress in the treatment of absence seizures in these populations.

## FUNDING INFORMATION

Trial funded by UCB Pharma.

## CONFLICTS OF INTEREST

Thomas Bast has received support from Bial, Desitin Arzneimittel GmbH, Eisai, GW Pharmaceuticals, Neuraxpharm, Novartis, Nutricia, Shire, Takeda, UCB Pharma, and Zogenix. Anne‐Liv Schulz, Florin Floricel, Diego Morita, Jody Cleveland, and Jan‐Peer Elshoff are salaried employees of UCB Pharma and have received stock or stock options from their employment.

## ETHICAL APPROVAL PUBLICATION STATEMENT

We confirm that we have read the Journal's position on issues involved in ethical publication and affirm that this report is consistent with those guidelines.

## Supporting information


AppendixS1

## Data Availability

Data from this trial may be requested by qualified researchers 6 months after product or indication approval in the USA and/or Europe, or global development is discontinued, and 18 months after trial completion. Investigators may request access to anonymized individual patient‐level data and redacted trial documents which may include: analysis‐ready datasets, study protocol, annotated case report form, statistical analysis plan, dataset specifications, and clinical study report. Before use of the data, proposals need to be approved by an independent review panel at www.Vivli.org and a signed data sharing agreement will need to be executed. All documents are available in English only, for a prespecified time, typically 12 months, on a password‐protected portal. This plan may change if the risk of reidentifying trial participants is determined to be too high after the trial is completed; in this case and to protect participants, individual patient‐level data would not be made available.
