# Supplementary material for: Efficacy and tolerability of brivaracetam monotherapy in childhood and juvenile absence epilepsy: An innovative adaptive trial design
Source: Epilepsia Open. 2022 Aug 4;7(4):588–97. doi: 10.1002/epi4.12628 (PMC9712476; doi:10.1002/epi4.12628)
Supplement: Supplementary file 1 — AppendixS1 [file EPI4-7-588-s001.docx]

## Supporting Information

## Supplemental methods

### Eligibility criteria

This trial will enroll patients 2-25 years of age and with a body weight of at least 9 kg. Patients from 2 to <4 years of age will not be included in Stage 1. Patients up to 25 years of age were included to also investigate BRV treatment of typical absence seizures in adults. Patients have to be diagnosed with CAE or JAE as defined by ILAE criteria. Patients from 2 to <4 years of age and patients with onset of absence seizures at <4 years of age must have a negative genetic test result for *GLUT1DS* mutations. Patients are enrolled if they are untreated with ASMs or pretreated for absence seizures with a maximum of two historical ASMs, but without ASM for at least five half-lives of the ASM before randomization into the trial.

Further inclusion criteria include: EEG evidence of bilateral synchronous, symmetric generalized paroxysmal spike waves (2.5-6.0 Hz) with normal background activity and with at least one electrographically recorded seizure lasting 3 seconds or more on a 1-hour EEG with hyperventilation while awake at visit 1 or on a historical EEG up to 12 weeks before enrollment; a history of clinically evident absence seizures occurring on at least 3 days per week in the 2 weeks before enrollment; no treatment with psychiatric drugs or on a stable dose for at least 2 weeks before randomization; and normal neurologic examination, head size, development, and cognition.

Patients are excluded if they have a history of nonfebrile seizures other than absence seizures, history of absence status epilepticus, history or presence of pseudo-seizures, history of major psychiatric disease, have participated in another study of an investigational drug or device within 30 days before informed consent, have use of the strong CYP2C19 inducer rifampicin/rifampin within 2 months before randomization, have use of strong CYP2C19 inhibitors (fluconazole, fluoxetine, fluvoxamine) within 1 week before randomization, have active suicidal ideation before trial entry, have a lifetime history of suicide attempt, or are pregnant or breastfeeding.

### Concomitant treatments

Use of any concomitant ASM is prohibited during the trial until the end of the RDW period. Use of concomitant ASMs (apart from felbamate and vigabatrin) is permitted during the down-titration period (blinded or unblinded). All non-ASMs are permitted throughout the trial, except for rifampicin/rifampin, fluconazole, fluoxetine, and fluvoxamine. Rescue medication is considered any treatment with a benzodiazepine (BZD); a BZD dose within 6 days after a previous BZD dose is prohibited. Ideally, rescue medication will not be administered during or within 4 days before the 24-hour EEGs. The use of substrates of alcohol dehydrogenase is prohibited.

### Compliance

The case report form will be used to record trial drug dispensing and return information on a by-patient basis and will serve as source documentation. Patients must return all unused trial drug and empty trial drug containers for assessment of drug accountability. If a patient is found to be persistently noncompliant (defined as <85% or >115% of prescribed dose), the sponsor, in conjunction with the investigator, will decide whether the patient should be withdrawn from the trial.

### Data collection

The trial sponsor or designee will be responsible for the data management of this trial including quality checking of the data. Monitors will perform ongoing source data verification, ensure protection of patient safety and rights, and trial conduct in accordance with the currently approved protocol and any other trial agreements, International Conference on Harmonization Good Clinical Practice (ICH GCP) guideline, and all applicable regulatory requirements. Source documents should be kept in a secure, limited access area.

### Monitoring

Safety will be monitored by the IDMC throughout the trial. Selected data will be reviewed periodically to detect as early as possible any safety concern(s) related to the trial drug so that investigators, trial participants, regulatory authorities, and Institutional Review Boards/Independent Ethics Committees will be informed appropriately and as early as possible.

### Ethical declarations

Written informed consent will be obtained from the patients or his/her parent/legal representative before trial participation. This trial will be conducted in compliance with the ICH GCP guideline and the Declaration of Helsinki; and with the Health Insurance Portability and Accountability Act for United States sites. The trial protocol (N01269 Amendment 1; September 3, 2020) has been approved by local Institutional Review Boards/Independent Ethics Committees, as defined in local regulations; any amendments will be similarly reviewed and approved. Patient confidentiality will be upheld throughout the trial, with all data identified only by patient number assigned at screening by the interactive web or voice response system.

### COVID-19

As a consequence of COVID-19, remote visits may be conducted and ad hoc patient contact may be warranted. Investigators and trial coordinators may use discretion when determining the need to perform a home visit (safety laboratory parameters, PK samples). Under coronavirus pandemic circumstances patients who cannot attend a protocol-defined trial visit required to be on-site will be discontinued.

**TABLE S1** Timeline of assessments

| **Trial period** | **Screening** | **BL** | **DB PC** | | **DB AT** | | | | | **RDW** | | | **DT** | **SFU** | **EDV** | **Unsch** |
| --- | --- | --- | --- | --- | --- | --- | --- | --- | --- | --- | --- | --- | --- | --- | --- | --- |
| Visit | V1 | V2 | V2a | V3 | V3a | V4 | TC | V5 | V5a | V6 | V7 | V7a | V8 | V9 |  |  |
| Week |  |  |  | W2 |  | W4 | W8 | W12 |  | W13 | W17 |  | End of DT | 2W after DT |  |  |
| Complete physical/ neurological examination | X | X |  | X |  |  |  | X |  | X | X |  | X |  | X | X |
| Brief physical/ neurological examination |  |  |  |  |  | X |  |  |  |  |  |  |  | X |  |  |
| C-SSRS | X | X |  | X |  | X |  | X |  | X | X |  | X | X | X |  |
| Prior concomitant medications and procedures | X | X |  | X |  | X | X |  |  | X | X |  |  | X | X |  |
| Vital signs; Body weight; Pregnancy test as appropriate | X | X |  | X |  | X |  |  |  | X | X |  |  | X | X |  |
| Hematology, serum chemistry, urinalysis | X |  |  | X |  | X |  |  |  | X | X |  |  | X | X |  |
| Endocrinology | X |  |  |  |  |  |  |  |  | X |  |  |  |  |  |  |
| 12-lead ECG | X |  |  | X |  |  |  |  |  | X | X |  |  | X | X | X |
| Recording of AEs | X | X | X | X | X | X | X | X | X | X | X | X | X | X | X | X |
| EEG (1 h awake EEG/hyperventilation) | X^a^ |  |  |  |  |  |  |  |  |  |  |  |  |  |  | X |
| EEG 24 h ambulatory with hyperventilation handout |  | X |  | X |  |  |  | X |  |  | X |  |  |  |  | X |
| EEG 24 h ambulatory collection |  |  | X |  | X |  |  |  | X |  |  | X |  |  |  |  |
| Trial drug collection and accountability |  |  |  |  | X | X |  |  |  | X |  | X | X |  | X | X |
| PedsQL |  | X |  | X |  |  |  | X |  |  |  |  |  |  | X |  |
| EpiTrack Junior |  | X |  | X |  |  |  | X |  |  |  |  |  |  | X |  |
| Dispense/collect patient diary | X | X |  | X |  | X |  | X |  |  |  | X | X |  | X |  |
| Blood sampling for serum/plasma PK levels |  |  |  | X |  |  |  |  |  | X | X |  |  |  |  |  |
| Abbreviations: AE, adverse event; AT, active treatment; BL, baseline; C-SSRS, Columbia Suicide Severity Rating Scale; DB, double-blind; DT, down-titration; ECG, electrocardiogram; EDV, early discontinuation visit; EEG, electroencephalogram; h, hour; PC, placebo-controlled; PedsQL, Pediatric Quality of Life; PK, pharmacokinetic; RDW, randomized withdrawal; SFU, safety follow-up; TC, telephone contact; Unsch, unscheduled; V, visit; W, week.  ^a^Not necessary if an absence seizure was recorded on an EEG conducted within 12 weeks before enrollment. | | | | | | | | | | | | | | | | |
